# Therapeutic efficacy of acupuncture on motor dysfunction in ischemic stroke patients with hemiplegia and its EEG characteristics: protocol for a randomized, sham-acupuncture controlled, assessor-and-statistician-blinded trial

**DOI:** 10.3389/fneur.2025.1653873

**Published:** 2025-09-26

**Authors:** Yue-hua Gu, Jun-xian Chen, Cui-na Yan, Li-wei Wang, Yi-ling Miu, Jian-xiang Li, Yi-yun Gu, Jie Xu, Ming Xia, Xiao-jing Zhang, Lu Yu

**Affiliations:** ^1^Comprehensive Department of Traditional Chinese Medicine, Putuo Hospital, Shanghai University of Traditional Chinese Medicine, Shanghai, China; ^2^Department of Neuroelectrophysiology, Putuo Hospital, Shanghai University of Traditional Chinese Medicine, Shanghai, China

**Keywords:** acupuncture therapy, ischemic stroke, limb dysfunction, electrophysiological mechanisms, randomized controlled trial, study protocol

## Abstract

**Background:**

Stroke is the second leading cause of death in the world, with high disability rate in survivors, among which ischemic stroke accounts for over 80% of the total. Limb dysfunction is a common neurological deficit symptom left by ischemic stroke. Timely rehabilitation therapy in the early recovery stage of stroke is important for neurological function improvement. Acupuncture therapy for “simultaneous treatment of phlegm and blood stasis,” selecting acupoints based on pathogenic characteristics of ischemic stroke, has been applied to treat stroke patients with hemiplegia in our center, and it has exhibited significant effects. However, high-quality clinical evidence of this acupuncture strategy is still lacking. Therefore, the present study aims to evaluate the efficacy and safety of acupuncture therapy in improving limb function of patients with recovery-stage ischemic stroke.

**Methods:**

This study will be a single-center, randomized, assessor-and-statistician-blinded, sham acupuncture-controlled clinical trial. After informed consent signing, 70 eligible patients with limb dysfunction in post-stroke recovery stage will be randomized into the treatment group or sham acupuncture group in a 1:1 ratio. The treatment course will last for 2 weeks, and the scale evaluation and EEG examination will be conducted before and after treatment. The primary outcome is the changes of limb function pre and post treatment using the modified Fugl-Meyer scale. Secondary outcomes include score changes in Berg Balance Scale, Fugl-Meyer Assessment Sensory Function Scale, Modified Ashworth Score, Barthel Index and TCM syndrome score pre and post treatment. Additionally, we will employ EEG to evaluate regulation effects of acupuncture on cortical neuronal excitability and functional connectivity across brain regions in stroke patients, and screen EEG-based biomarkers with predictive value.

**Discussion:**

This study aims to evaluate the efficacy and safety of acupuncture therapy in stroke patients with hemiplegia, and to explore electrophysiological mechanisms of the therapeutic effects. The study will provide high-quality clinical evidence for the application of acupuncture therapy with acupoint combination based on the TCM theory in neurological rehabilitation for ischemic stroke.

**Clinical trial registration:**

itmctr.ccebtcm.org.cn, identifier ITMCTR2025001171.

## 1 Introduction

, Stroke is one of the major diseases threatening human health in the world. According to statistics, the prevalence of stroke in China had reached 26 million by 2021. Stroke is characterized by a prolonged disease course, high medical cost, and high recurrence rate, which seriously affect patients’ life quality and impose a significant economic burden on society. With the improvement of medical standards in recent years, the mortality rate of stroke has decreased (1), however, its disability rate remains as high as 70 to 80% (1, 2). Most patients suffer from varying degrees of neurological impairment, such as hemiplegia, spasticity, dysphagia, sensory dysfunction, cognitive deficits, hemianopsia, and aphasia, among which hemiplegia is the most common post-stroke sequela, significantly impacting patients’ physical and mental states. If not treated timely and effectively, it will lead to permanent disability. The occurrence of neurological deficit symptoms is tightly associated with neuronal damage, impaired vascular regeneration, disruption of interhemispheric connections and disturbance of synaptic activities in the brain (3).

Rehabilitation therapy is the primary method to restore impaired limb function in stroke patients by enhancing neuronal activities, increasing postsynaptic excitability, and promoting the regeneration of dendritic spines and the recovery of myelin sheaths (3). The conventional rehabilitation for stroke primarily includes neuromuscular electrical stimulation, physical therapy, occupational therapy, and rehabilitation robots (4). However, the high cost and cumbersome training methods make it difficult for patients to adhere to the therapy in long term, thus presenting limited effectiveness (5). In China, acupuncture experienced a long history over 2,000 years and has been widely applied in the treatment of limb dysfunction after stroke (6). Owing to its simplicity of operation, effectiveness, and safety, acupuncture may serve as an alternative for improving post-stroke sequelae with minimal adverse effects (7). Studies have suggested that acupuncture, as a form of somatosensory stimulation, can induce plastic changes among different representations in the motor cortex, modulate interhemispheric competitive inhibition, and improve cerebral cortical function (8, 9). Acupuncture applied to the unilateral limb can stimulate neuronal excitability in bilateral brain regions, increase cerebral blood flow, and enhance the consistency of neuronal activity in the motor area of the affected hemisphere (10). It has been reported that bilateral needling method of hand-foot 12 needles can improve the motor recovery post-stroke and modify cerebro-cerebellar voxel-mirrored homotopic connectivity (11). Scalp acupuncture therapy combined with exercise therapy can significantly improve stroke patients’ ability to participate in daily activities (12). However, the acupoint selection in these studies has failed to integrate with the pathogenic characteristics of stroke in TCM, thus not fully leveraging the advantages of syndrome differentiation in acupoint selection. Moreover, the mechanisms underlying the therapeutic effects of acupuncture for hemiplegia remain unclear, lacking the support of objective evidence, which limits the development and application of acupuncture in stroke rehabilitation to some extent.

According to TCM, phlegm and blood stasis are two major pathogenic products contributing to the occurrence of ischemic stroke (13). Studies indicate that the predominant TCM syndrome types for stroke are wind-phlegm obstructing collaterals syndrome and qi deficiency with blood stasis syndrome. Manifestations of blood stasis and phlegm turbidity syndromes are particularly prominent during stroke recovery (14) and positively correlate with worsening neurological deficits (15). The acupoints employed in acupuncture treatment are mainly distributed along the Twelve Regular Meridians, and they exert different therapeutic effects based on the pathways of meridians and functional characteristics of the zang-fu organs they are associated with. In TCM, it is believed that the Yangming Meridian is abundant in both qi and blood, and it nourishes the sinews and tendons. Acupuncture applied to this meridian can promote the qi flow of the Yangming Meridian, supplement qi, and tonify blood, contributing to the recovery of limb function in patients with hemiplegia (16). Additionally, TCM holds that “the spleen is the source of phlegm,” acupuncture applied to the Spleen Meridian can strengthen functions of the spleen and stomach and alleviate the pathological accumulation of phlegm-turbidity in the meridians (17). The Shaoyang Meridian, situated in the half-exterior-half-interior position of the body, functions as a pivot that regulates the flow of qi, disperses blood stasis to promote circulation, and unblocks the meridians and collaterals (18). Therefore, based on TCM meridian theory and stroke pathogenic characteristics, we formulate the therapeutic principle of “simultaneous treatment of phlegm and blood stasis” and select acupoints comprising Jianyu (LI5), Quchi (LI11), Hegu (LI4), Waiguan (SJ5), Zusanli (ST36), Fenglong (ST40), Xuehai (SP10), Sanyinjiao (SP6), and Xuanzhong (GB39) for stroke treatment. By using the specialized acupuncture method featuring acupoint combinations for phlegm and blood stasis syndromes, our center has achieved remarkable therapeutic outcomes in post-stroke patients with hemiplegia. Therefore, it is necessary to conduct a standardized, high-quality clinical trial to evaluate the efficacy of acupuncture in improving limb function in stroke patients with hemiplegia and to investigate the related mechanisms contributing to its therapeutic effects.

Electroencephalogram (EEG) is an important method for assessing brain function and widely used in clinical practice. It tracks and records the tiny electrical signals produced by cortical neuronal activity in real time by placing electrodes on the scalp. Brainwaves in different frequency bands reflect synchronized neuronal activities across distinct brain regions (19). Since EEG has a temporal resolution in milliseconds, it can capture changes in neuronal electrical activity more rapidly than MRI and is highly sensitive to abnormalities in brain metabolism and neural function (20). Due to its simplicity and non-invasive nature, EEG has been applied to evaluate the therapeutic effect after intervention for brain diseases. As evidenced by EEG, in stroke patients with better clinical outcomes, the healthy hemisphere tended to compensate for the affected side, and kinematic and EEG metrics were reported to be relevant for evaluating early post-stroke motor recovery (21). Additionally, event-related desynchronization (ERD) during action observation, reflecting reduced coordination of neural activity, was validated as an early predictor of motor recovery in subcortical stroke (22). These metrics provide important prognostic information that can be used to optimize treatment strategies. Herein, we design a randomized, assessor-and-statistician-blinded, parallel-controlled clinical trial, for assessing the efficacy and safety of acupuncture therapy, featuring acupoint combination for “simultaneous treatment of phlegm and blood stasis,” in treating limb dysfunction in ischemic stroke patients with hemiplegia. We will employ functional scales to evaluate patients’ clinical outcomes and use EEG to capture neurophysiological changes after acupuncture, including neural excitability and brain functional connectivity. Further, EEG metrics will be linked with clinical outcomes, demonstrating its multiple values in efficacy assessment, mechanism interpretation, and prognosis prediction.

## 2 Methods and analyses

### 2.1 Study design

This is a single-center, assessor-and-statistician-blinded, randomized, sham acupuncture-controlled clinical trial. The study aims to evaluate the efficacy and safety of acupuncture therapy, featuring acupoint combination for “simultaneous treatment of phlegm and blood stasis,” in treating ischemic stroke patients with hemiplegia. The study has been registered on itmctr.ccebtcm.org.cn (Registration No. ITMCTR2025001171). SPIRIT checklist is shown in Supplementary file 1.

**Figure 1:**
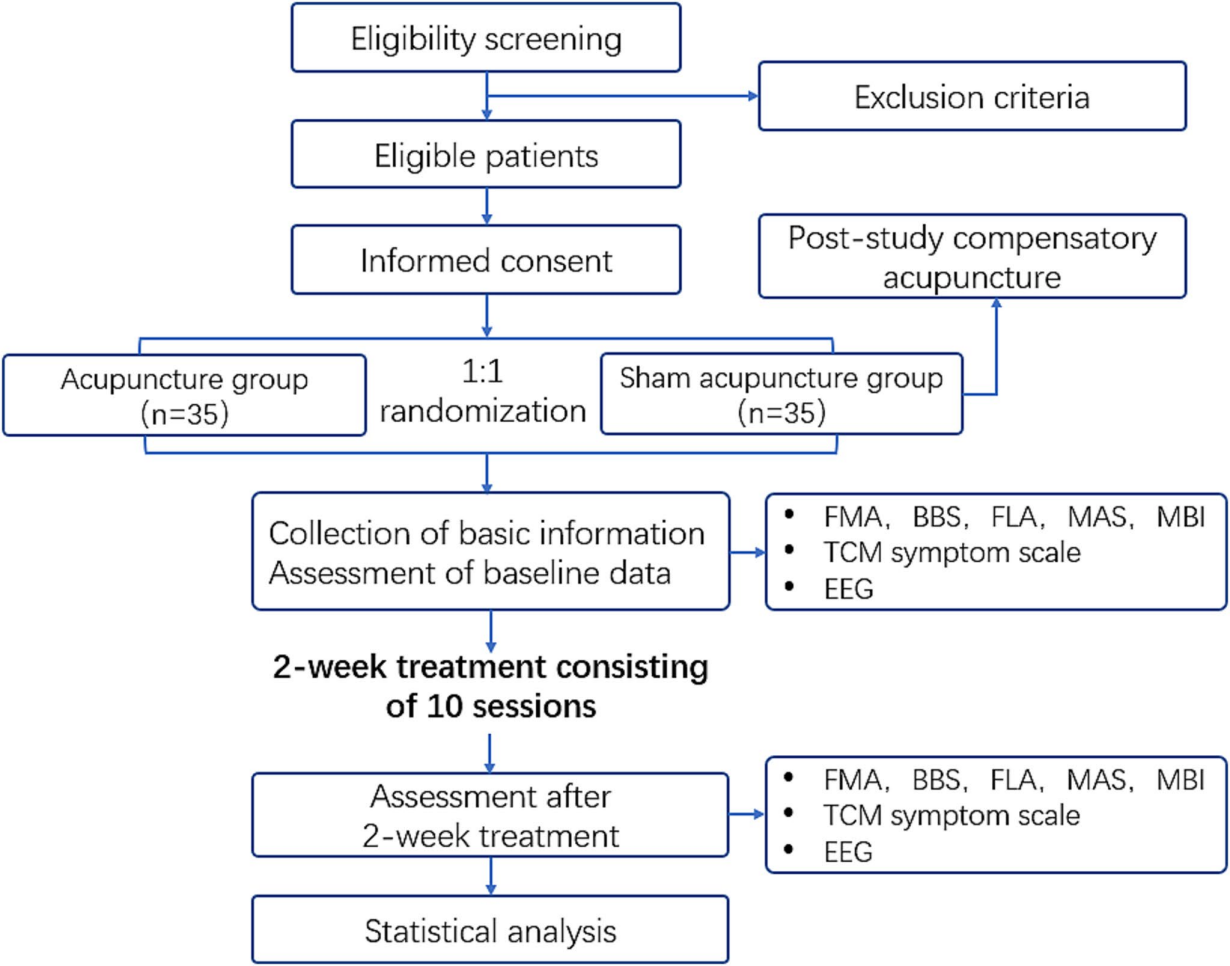
Flow diagram of the study.

The study will recruit 70 eligible ischemic stroke patients with unilateral limb dysfunction who meet the inclusion criteria. After signing informed consent, the participants will be randomized into acupuncture group or sham acupuncture group in a 1:1 ratio. All participants will receive a two-week treatment. Basic information will be collected before treatment (Day −1 to 0), and electroencephalogram (EEG) examination and scale evaluation will be conducted before (Day −1 to 0) and after the two-week treatment (Day 15). Allocation information will be confidential to assessors. The study process is shown in Figure 1 and the timeline for enrolment, intervention and assessment is shown in Table 1. The study will be started in June 2025 and complete recruitment by June 2028. The entire study is expected to be finalized by December 2028.

**Table 1:** Participant timeline.

	Enrolment and baseline	Intervention period (2 weeks, 5 times per week)				
Timepoint	Before treatment	Day 1–5	Day 6–7	Day 8–12	Day 13–14	Day 15
Patients						
Recruitment and screening	√					
Signed informed consent	√					
Randomization	√					
Allocation	√					
Interventions						
Acupuncture		√		√		
Sham acupuncture		√		√		
Outcome measures						
FMA	√					√
BBS	√					√
FMA-S	√					√
MAS	√					√
MBI	√					√
TCM symptom score	√					√
EEG	√					√
Patients’ compliance	√	√	√	√	√	√
Safety assessment						
Adverse events	√	√	√	√	√	√
ECG	√	√	√	√	√	√

FMA, short form Fugl-Meyer assessment for motor function; BBS, the Berg balance scale; FMA-S, the sensory scale of the Fugl-Meyer assessment; MAS, the modified Ashworth scale; MBI, the modified Barthel index; EEG, Electroencephalogram data collection and processing; ECG, Electrocardiogram examination.

### 2.2 Patient recruitment

Patient screening and recruitment will be conducted from June 2025 to June 2028 in Traditional Chinese Medicine Comprehensive Ward of Shanghai Putuo District Central Hospital. Ischemic stroke patients with unilateral limb dysfunction who meet the inclusion criteria will be fully informed about the objective, content, benefits, and potential risks of the study, and be randomly assigned to different groups after informed consent.

### 2.3 Diagnostic criteria

The diagnostic criteria for Western medicine refer to the “Chinese Guidelines for Diagnosis and Treatment of Acute Ischemic Stroke 2023” (23), while the diagnostic criteria for TCM syndrome differentiation in stroke refer to the classification criteria for phlegm syndrome and blood stasis syndrome outlined in the “Diagnostic and Efficacy Evaluation Criteria for Stroke” (24).

### 2.4 Inclusion criteria

1. Patients who meet diagnostic criteria of Western medicine for ischemic stroke, combined with CT or MRI;
2. Patients who meet diagnostic criteria for phlegm and blood stasis obstructing meridians in TCM syndrome;
3. Aged between 30 and 80, inclusive;
4. First occurrence, or those with a previous stroke but without sequelae;
5. 1 week to 3 months after the onset of stroke;
6. Lesion in one cerebral hemisphere with unilateral hemiplegia, Brunnstrom stage II-IV;
7. Right handedness;
8. Patients with clear minds, clinically stable conditions, and limb dysfunction caused by stroke.

### 2.5 Exclusion criteria

1. Patients who suffered from transient ischemic attack, hemorrhagic stroke, and asymptomatic cerebral embolism;
2. Patients with severe multi-system diseases and mental disorder;
3. Patients with severe consciousness disorders, cognition impairment, and post-stroke depression;
4. Excluding factors that interfere with EEG signals (such as receiving transcranial magnetic stimulation within 1 month) (25);
5. Patients with severe head skin injuries;
6. Patients with severe needle phobia or allergic to acupuncture needles.

### 2.6 Dropout criteria

1. Patients who fail to cooperate during treatment, voluntarily request to withdraw from the trial, or discontinue participation;
2. Patients who experience unbearable pain during acupuncture and are unable to continue treatment;
3. Patients who develop severe complications during treatment and are no longer suitable for further treatment.

### 2.7 Sample size calculation

Sample size for this study is estimated according to the formula for the comparison of means in a randomized controlled trial. Referring to the relevant literatures (26, 27), we estimate the pooled standard deviation (*σ*) of the treatment group and the control group to be 7.81, and the difference between the means of the two groups (*δ*) to be 6.59. We set the significant level (*α*) at 0.05, the Type II error rate (*β*) at 0.1, and the statistical power (1-β) at 0.9. After substituting the values into the formula, calculated sample size per group requires 30 cases. Considering a 15% dropout rate, a total of 70 cases will be enrolled.


$$n=\frac{2{\left(z_{\alpha }+z_{\beta }\right)}^{2}*\sigma^{2}}{\delta^{2}}$$

### 2.8 Randomization and allocation concealment

The grouping will be performed using a completely random method. The random allocation sequence will be generated through an online randomization tool Med Sci (http://tools.medsci.cn/rand) and managed by an independent researcher. Stroke patients who meet the inclusion criteria will be randomized into the acupuncture group or the sham acupuncture group in a 1:1 ratio after signing the informed consent. Cards printed with information on the allocated groups will be placed in opaque envelopes. Patients will receive envelopes sequentially according to the order of inclusion. An acupuncturist will open envelopes prior to treatment and conduct the corresponding treatment. The randomization process will be carried out by an independent researcher who is not involved in the recruitment, evaluation, and intervention.

### 2.9 Blinding

This study will employ blinding in efficacy assessment and statistical analysis. Since sham acupuncture will be used as the control, it is difficult to blind participants and acupuncturists. Additionally, the grouping information will be blinded to efficacy assessors and statistical analysts. Acupuncturists will administer acupuncture therapy in accordance with the treatment plans corresponding to the random numbers. Patients will be separated to prevent communication. Researchers responsible for scale evaluation and EEG analysis will not participate in the grouping or intervention processes. Besides, statistical analyst will not be involved in the implementation of the study to avoid selective bias produced by objective factors.

### 2.10 Intervention

#### 2.10.1 Conventional treatment

Eligible patients will be randomized into the acupuncture group or sham acupuncture group for a two-week treatment. During the study period, all patients will receive Western medicine treatment following “Chinese Guidelines for Diagnosis and Treatment of Acute Ischemic Stroke 2023” (23), including anti-platelet aggregation, improving cerebral circulation, reducing blood pressure, lowering blood glucose, lowering lipid, etc. Simultaneously, patients will undergo routine rehabilitation treatment in accordance with the “China Guidelines for Stroke Rehabilitation” (28), which includes physical therapy treatments, such as proper limb positioning, joint range-of-motion training, muscle strengthening training, balance and coordination training, standing training, and gait training, as well as occupational therapy, involving fine motor training and activities of daily living training. The rehabilitation programs will be set based on individual conditions and carried out five times per week for 2 weeks, and each rehabilitation treatment session will last for approximately 1 h.

#### 2.10.2 Acupuncture treatment

**Figure 2:**
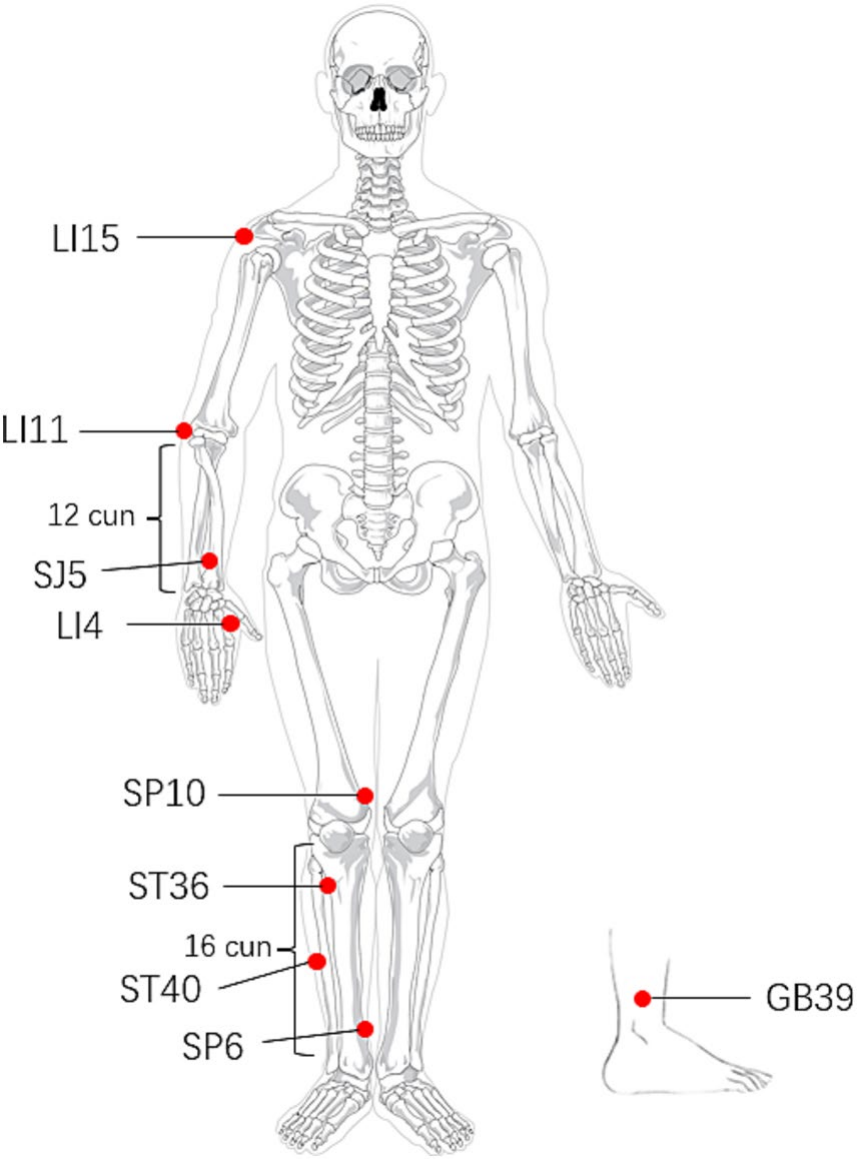
Location of acupoints.

Based on previous treatment experience, the acupuncturists will select combined acupoints on the affected side of stroke patients, guided by the TCM theory of “simultaneous treatment of phlegm and blood stasis,” which include Jianyu (LI15), Quchi (LI11), Hegu (LI4), Waiguan (SJ5), Zusanli (ST36), Fenglong (ST40), Xuehai (SP10), Sanyinjiao (SP6), and Xuanzhong (GB39). The acupoint location conforms to “nomenclature and location of meridian points” (GB/T12346-2021) issued in 2021. Acupuncture therapy will be administered by a licensed acupuncturist with over 5 years of experience, who has undergone uniform training prior to the onset of the study. The patients will take the supine position, fully exposing acupuncture site. After disinfection with 75% medical alcohol, the acupuncturist will employ disposable sterile stainless-steel needles (0.25 × 40 mm, Huatuo, Suzhou, China) for the treatment. According to different acupoints, the acupuncturist will insert needles vertically and manipulate them in turn by twirling, lifting, and thrusting for 1 min until the *De qi* sensation is achieved (a compositional sensation including soreness, numbness, distention, and heaviness). After that, the needle will be retained for 30 min. The localization of the acupoints is presented in Table 2 and Figure 2. The treatment will be administered for 5 consecutive days from Monday to Friday, followed by a 2-day weekend, making it a 10-day treatment course in total, and the duration of each session will be 30 min. The temperature in the treatment room remain above 25°C during the treatment for ensuring patients’ comfort and safety.

**Table 2:** Location of acupoints.

Acupoints	Meridians	Locations	Insert angle	Insert depth
Jianyu (LI15)	Large Intestine Meridian of Hand-Yangming	At the inferior border of the acromial end of the clavicle, between the acromion and the greater tuberosity of the humerus, in the central upper part of the deltoid	90°	1 cun
Quchi (LI11)	Large Intestine Meridian of Hand-Yangming	With the elbow flexed to 90°, at the midpoint of the line connecting the lateral border of the elbow flexion crease and the lateral epicondyle of the humerus	90°	1 cun
Hegu (LI4)	Large Intestine Meridian of Hand-Yangming	At the midpoint on the radial side of the 2^nd^ metacarpal bone between the 1^st^ and 2^nd^ metacarpals	90°	1 cun
Waiguan (SJ5)	Sanjiao Meridian of Hand-Shaoyang	At 2 cun proximal to the distal wrist crease, on the midpoint of the line connecting the ulna and radius	90°	1 cun
Zusanli (ST36)	Stomach Meridian of Foot-Yangming	At 3 cun distal to Dubi (ST35) along the line connecting Dubi and Jiexi (ST41), one finger-breadth lateral to the anterior border of the tibia	90°	1.5 cun
Fenglong (ST40)	Stomach Meridian of Foot-Yangming	At 8 cun superior to the tip of the lateral malleolus, and two finger-breadths lateral to the anterior crest of the tebia	90°	1.5 cun
Xuehai (SP10)	Spleen Meridian of Foot-Taiyin	Flex knee 90°, at 2 cun above the medial end of the patella’s top edge, at the peak of the inner thigh muscle bulge	90°	1 cun
Sanyinjiao (SP6)	Spleen Meridian of Foot-Taiyin	At 3 cun above the medial malleolus apex, along the posterior depression adjacent to the tibial medial border	90°	1 cun
Xuanzhong (GB39)	Gallbladder Meridian of Foot-Shaoyang	At 3 cun above the lateral melleolus apex, along the anterior border of the fibula	90°	1 cun

#### 2.10.3 Sham acupuncture treatment

**Figure 3:**
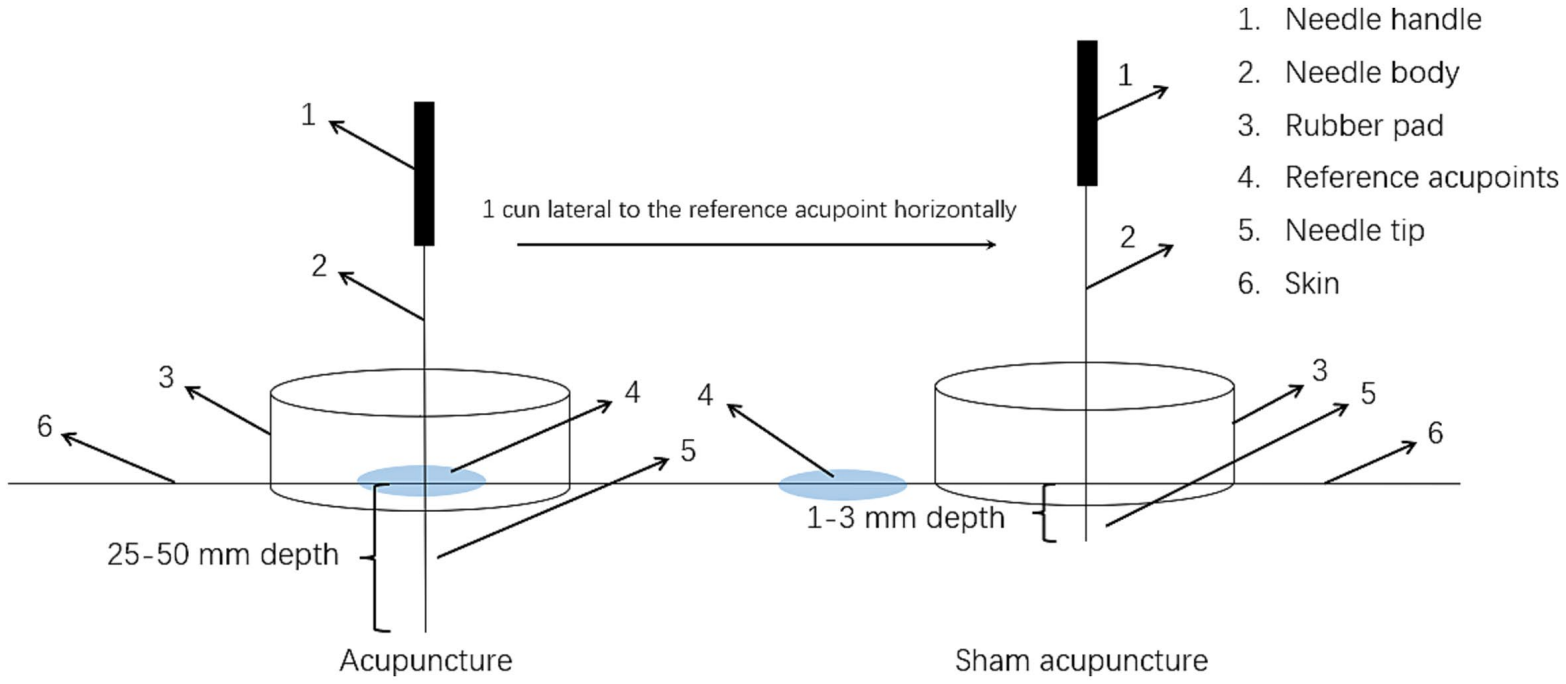
Diagram of the acupuncture and sham acupuncture.

The sham acupuncture method will be employed as a parallel control for the acupuncture treatment group. The operation of the sham acupuncture will be defined by three elements (29): (1) the acupuncture points are located 1 cun lateral to the reference acupoints at the horizontal level, and needling these positions will not affect the therapeutic efficacy for stroke; (2) superficial insertion (depth of 1–3 mm); (3) no manipulation after needle insertion to avoid achieving the *De qi* sensation (Figure 3). A rubber pad will be placed on the skin of corresponding acupuncture sites of the two groups to alleviate patients’ fear of needling. Importantly, it simulates the sensation of real acupuncture manipulation, keeping the needle upright on the skin without actual skin penetration in the sham acupuncture group. Patients in the sham acupuncture group will receive 2 weeks of compensatory acupuncture treatment after the observation period.

### 2.11 Basic information collection

Basic demographic and medical history data from eligible patients will be collected using a self-designed questionnaire, including gender, age, education, marital status, residence, occupation, as well as smoking/alcohol use, family stroke history, underlying diseases, and medication history. Basic information and baseline assessments will be recorded and gathered post random grouping.

### 2.12 Outcome measures

#### 2.12.1 Primary outcome

The primary outcome is the change in limb motor function in stroke patients before and after treatment, assessed using the simplified Fugl-Meyer Assessment (FMA) scale. Limb motor function impairment will be quantified by evaluating upper/lower extremity functions, balance, sensation, and joint range of motion. The scale comprises 50 items (33 for upper limbs, 17 for lower limbs), with higher scores indicating better functional activity.

#### 2.12.2 Secondary outcome

##### 2.12.2.1 Balance ability

The static and dynamic balance ability of patients will be assessed using the Berg Balance Scale (BBS). The scale includes 14 items and uses a 5-point rating system. Each item is scored 0–4 according to the patient’s performance, for a total of 56 points, with higher scores indicating better balance ability.

##### 2.12.2.2 Sensory function

The degree of sensory impairment will be assessed using the sensory scale of the Fugl-Meyer Assessment (FMA-S). The scale evaluates light touch and proprioception with 12 items (2 points each), totaling 24 points, with higher scores indicating better sensory function.

##### 2.12.2.3 Muscle tone

Limb muscle spasticity will be evaluated using the Modified Ashworth Scale (MAS) by assessing the resistance encountered during passive muscle stretching. The scale has 6 levels, ranging from no increase in muscle tone to rigidity. Level 0 indicates normal joint mobility, while level 4 indicates the most severe spasticity, with the joint immovable.

##### 2.12.2.4 Activities of daily living

The impact of limb dysfunction on daily living activities will be assessed using the Modified Barthel Index (MBI). The scale includes 10 items, each scored 0–10, totaling 100 points, with higher score indicating better independence in daily life for stroke patients.

##### 2.12.2.5 TCM syndrome score

TCM syndrome scoring scale is drawn in accordance with the “Diagnostic and Efficacy Evaluation Criteria for Stroke” (24). The scale quantifies clinical symptoms and signs related to phlegm-turbidity and blood stasis in stroke patients through TCM syndrome scoring, totaling 14 items, with higher score indicating more severe symptom.

##### 2.12.2.6 EEG data collection

**Figure 4:**
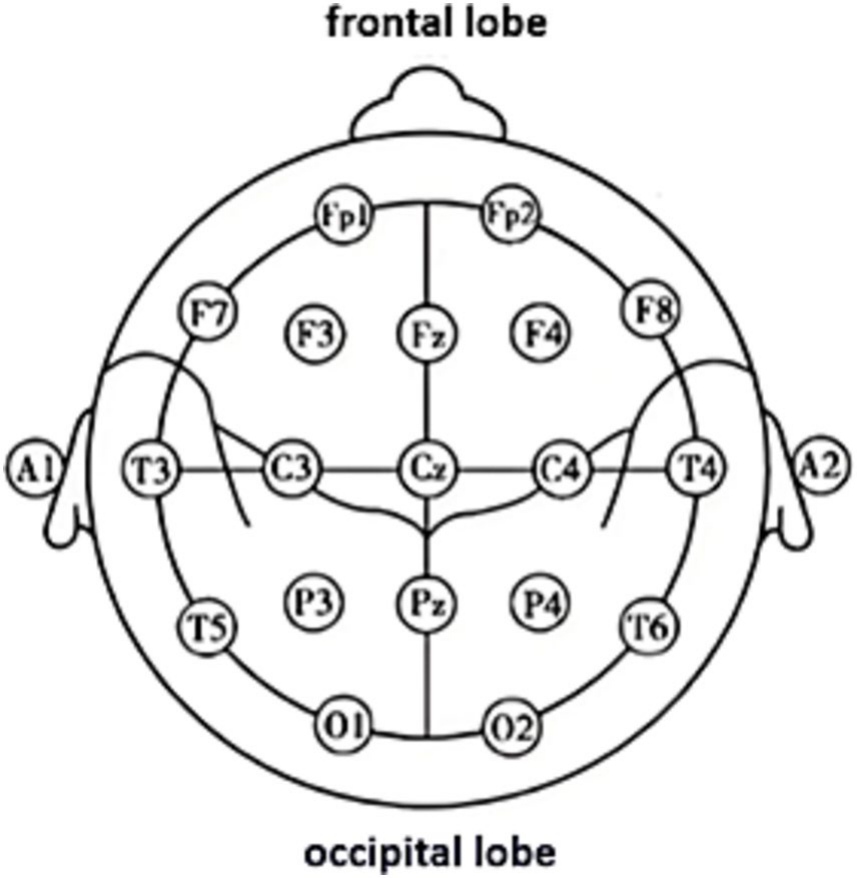
Electrode positions in the international 10–20 system.

Patients will undergo EEG examination before and after treatment, respectively. EEG recording will be conducted in a dimly lit and quiet environment to avoid visual and auditory distractions. Patients are required to avoid caffeinated beverages, sedatives, and stimulants for three days prior to the examination. They should also ensure 7.5 h of sleep before the test and avoid fatigue, hunger, and hypoglycemia. During the examination, they should stay conscious, keep their eyes closed, remain relaxed, and refrain from feeling tense or anxious. The study will employ the 32 U-type digital electroencephalogram/brain electrical activity mapping (EEG/BEAM) instrument, with electrodes placed according to the international 10–20 system (refer to Figure 4). In the unipolar lead method, bilateral earlobes (A1, A2) are selected as the reference electrodes to record the potential difference between the active electrodes and the reference electrodes. The lead arrangement is as follows: FP1-A1, FP2-A2, F3-A1, F4-A2, C3-A1, C4-A2, P3-A1, P4-A2, O1-A1, O2-A2, F7-A1, F8-A2, T3-A1, T4-A2, T5-A1, T6-A2. The bipolar lead method records the potential difference between two adjacent electrodes, anterior and posterior, in symmetrically paired regions of the left and right hemispheres. The lead arrangement is as follows: FP1-F3, F3-C3, C3-P3, P3-O1, FP2-F4, F4-C4, C4-P4, P4-O2, FP1-F7, F7-T3, T3-T5, T5-O1, FP2-F8, F8-T4, T4-T6, T6-O2. Parameter settings: sensitivity 10 μv/mm, time base 30 mm/s, time constant 0.3, high-pass filter 70 Hz, low-pass filter 0.53 Hz. Routine eye-opening/closing evoked test will be conducted during recording, with a duration of 20 min. An independent researcher will analyze EEG data, calculating the frequency and amplitude of background brainwave patterns in each hemisphere and abnormal waveforms in lesioned regions. EEG data will be collected using the XLTEK neuroworks EEG system.

##### 2.12.2.7 EEG data processing

EEG signals collected from electrodes placed at different head regions are filtered to remove interference signals with frequencies below 0.5 Hz and above 30 Hz. Independent component analysis (ICA) is applied to eliminate non-cerebral interferences from the EEG data, such as electrooculography (EOG) artifacts and electromyography (EMG) signals. After amplified, the processed signals are input into a computer’s analog-to-digital converter (ADC), where the waveform signals are transformed into digital information and stored in the computer’s memory. Fast Fourier Transform (FFT) will analyze voltage variables at different time phases to process the power spectra across various frequency bands. The full-band spectral power plot is displayed with a frequency resolution of 1 Hz per step, and power plots are generated for the four frequency bands: delta (1–4 Hz), theta (4–8 Hz), alpha (8–13 Hz), and beta (13–30 Hz). The grayscale images will be converted into color bands, their grayscale levels will be numerically quantified, and a grayscale scale will be generated for analysis using BESA (Brain Electrical Source Analysis, BESA) software.

##### 2.12.2.8 Coherence analysis

Coherence is an index used in EEG analysis to measure signal synchrony between two brain regions, reflecting the degree of functional connectivity between them. The coherence C*_xy_* (*f*) between two EEG signals *x* and *y* from different electrodes at frequency *f* is calculated using the following formula:


$$C_{\mathrm{xy}}(f)=\frac{|P_{\mathrm{xy}}(f)|2}{P_{\mathrm{xx}}(f).P_{\mathrm{yy}}(f)}$$

*P_xx_* (*f*) and *P_yy_* (*f*) represent the power spectral densities of signals *x* and *y*, respectively, while *P_xy_* (*f*) is the cross-spectral density between *x* and *y*, all of which are estimated using the Welch method. Coherence ranges from 0 to 1, with higher values indicating greater similarity in phase and amplitude between EEG signals, among which, a value of 1 indicates complete synchronization, while 0 indicates complete desynchronization. This study will calculate coherence in the alpha and beta bands, designating the M1 region (primary motor cortex) on the ipsilesional or contralesional side of the lesion as the seed region, and will calculate coherence values between the seed region and all other regions. In the 32-electrode layout of the international 10–20 system, the M1 seed region is defined as C3 (for the left hemisphere M1) or C4 (for the right hemisphere M1). When the infarct lesion is located in the right hemisphere, the data will be flipped along the midline to standardize the analysis hemisphere. For normally distributed data, two independent-samples t-test will be conducted, while for non-normally distributed data, non-parametric Wilcoxon test will be applied to compare Coherence differences between the two groups. Additionally, Cohen’s *d* will be calculated to quantify the effect size, and the Bonferroni correction will be employed to control the false positive rate associated with multiple comparisons.

### 2.13 Safety assessment

Safety will be assessed by observing and recording adverse events and conducting electrocardiogram (ECG) examination. Common adverse events associated with acupuncture therapy include bleeding, needle fainting, subcutaneous hematoma, and pain. The acupuncturist responsible for treatment needs to evaluate the severity of adverse events and promptly record and report the occurrence time, symptoms, duration, severity, treatment measures taken, and resolution time of such events. The grading for the severity of adverse events is categorized into three levels: grade 1 for mild, grade 2 for moderate, and grade 3 for severe or medically significance. Patients experiencing severe adverse events should stop acupuncture therapy, receive timely care, and the events must be reported to the Ethics Committee of Shanghai Putuo District Central Hospital within 24 h.

### 2.14 Data management

The case report form of each patient will be timely filled out by an independent researcher, with all data accurately recorded. Patients’ medical histories, original data, and CRF tables will be stored in the clinical research office. Data will be entered into an EXCEL sheet using a double-entry method, followed by cross-check for data consistency. Any discrepancies found will require a recheck and correction of the abnormal values based on the CRF and original data. The identified database will be converted into the SPSS statistical software format and locked for data analysis by a qualified statistician.

### 2.15 Measures to improve compliance

1. Patients will be followed up during the treatment course to closely monitor their compliance with the treatment.
2. Patients in the sham acupuncture group will receive compensatory therapy after the observation period.

### 2.16 Statistical analysis

All data will be analyzed using SPSS 27.0 statistical software. In accordance with the intention-to-treat (ITT) principle, patients who have received at least one treatment session will be included in the analysis. Basic information, baseline data, and efficacy evaluation will be analyzed using both the full analysis set (FAS) and per-protocol set (PPS). In case of inconsistent results between the two datasets, further analysis and evaluation will be conducted. Missing data will be imputed using multiple imputation method. For continuous outcomes, post-treatment data are presented as mean±SD if normally distributed with equal variances; otherwise, they are presented as median (IQR). ANCOVA will be used to adjust for baseline values and confounders (e.g., age, gender, revascularization therapy status), with adjusted mean differences and 95% CIs reported. For categorical data, frequencies and percentages will be presented, and potential confounders will be screened via univariate analysis before constructing stepwise logistic regression models to adjust for confounders and report odds ratios with 95% CIs, followed by validation of the model’s goodness of fit. Further, patients will be grouped based on infarction location to analyze EEG indicators in each subgroup. Receiver operating characteristic (ROC) curve analysis will be conducted to evaluate the predictive value of EEG as an efficacy indicator for acupuncture response, with the area under the curve (AUC) and 95% CI reported. For correlation analysis, Pearson correlation will be applied to normally distributed continuous data, while Spearman correlation will be used for ordinal data. All data analysis will be conducted using two-tailed tests with a significant level of 5%.

### 2.17 Ethical issues

The trial research protocol adheres to the principles of the Declaration of Helsinki and has been approved by the Ethics Committee of Shanghai Putuo District Central Hospital (PTEC-A-2024-46-2). In the case of any changes to the study protocol, researcher will promptly report the modifications to the Ethics Committee. Before the study begins, researcher will undergo informed consent training and draft an informed consent form to ensure that participants are fully informed about the study process.

## 3 Discussion

Since ischemic stroke poses a severe threat to health, thrombolysis and thrombectomy after acute stroke can effectively reduce stroke-related mortality and disability rates (30). However, the strict time window for these treatments limits stroke patients’ therapeutic benefits (31). Most patients experience varying degrees of neurological deficits after acute stroke, with approximately 50–80% developing limb motor dysfunction related to ischemic lesion during recovery period, which indicates a significant demand for post-stroke functional rehabilitation (32). The Stroke Roundtable Consortium defines the first 24 h post-stroke as the hyperacute phase, the first 7 days as the acute phase, the first 3 months as the early sub-acute phase, 4–6 months as the late sub-acute phase, and beyond 6 months as the chronic phase (33). In the early post-stroke period, a series of neuronal plasticity-enhanced mechanisms trigger dendritic growth, axon sprouting, and the formation of new synapses (34). The “proportional recovery rule” suggests that patients can recover approximately 70% of lost function within 3–6 months post stroke (35), though this proportion varies with individual differences, lesion location and severity, as well as the effectiveness of rehabilitation measures. Therefore, seizing the critical time window for post-stroke rehabilitation and implementing effective rehabilitation measures are crucial for restoring impaired neural functions, improving disease prognosis, and enhancing patients’ quality of life.

In TCM, phlegm turbidity and blood stasis are considered two major pathogenic factors contributing to ischemic stroke, persisting throughout the entire course of the disease (36). Therefore, guided by TCM theory and prior treatment experience, this study primarily selected acupoints on the Hand and Foot Yangming Meridians and the Foot Taiyin Spleen Meridian for acupuncture treatment, aiming to tonify qi, activate blood circulation, resolve phlegm, and dredge the meridians. Additionally, Waiguan on the Hand Shaoyang Sanjiao Meridian is selected to regulate qi movement and unblock the meridians, while Xuanzhong on the Foot Shaoyang Gallbladder Meridian is chosen to strengthen tendons and bones, and nourish the brain and marrow. The selection and combination of these acupoints align with the pathogenesis characteristics of stroke, reflecting the differentiation and treatment feature of “simultaneous treatment of phlegm and blood stasis.” Currently, there are few reports on RCTs that evaluate the therapeutic efficacy of acupuncture for ischemic stroke using both subjective and objective indicators, aiming to assess the syndrome differentiation-based acupoint selection in TCM, and to explore its underlying mechanisms. Herein, guided by the SPIRIT principle, we designed a single-center, randomized, assessor-and-statistician-blinded, sham acupuncture-controlled clinical trial to evaluate the efficacy of the “simultaneous treatment of phlegm and blood stasis” acupuncture therapy in improving limb motor function in ischemic stroke patients with hemiplegia. We used functional assessment scales combined with EEG to evaluate the efficacy, and explored its potential electrophysiological mechanisms by recording EEG activity. In this study, we have set the intervention time for acupuncture treatment to be between 1 week and 3 months post-stroke. After the 7-day acute phase, stroke enters the recovery phase, during which patients’ physical conditions stabilize, vital signs remain relatively steady, enabling them to tolerate and adapt to various rehabilitation treatments. Based on neurorehabilitation theory, active rehabilitation within 3 months post-stroke, defined as early recovery, is more conducive to propelling patients into the fast lane of functional recovery. And we will administer 10 acupuncture sessions (5 consecutive days per week for 2 weeks) to stroke patients with limb dysfunction during the early recovery phase, aiming to capture the optimal window for post-stroke rehabilitation. Clinical observation indicates that 10 sessions of acupuncture can lead to a certain degree of improvement in limb motor function (11). Moreover, an appropriate treatment course enhances patients’ compliance and cooperation. Given varying degrees of disease severity, some patients may choose to continue rehabilitation after the 2-week treatment. Thus, we did not schedule a follow-up assessment after the end of the treatment course. Furthermore, ischemic and hemorrhagic strokes exhibit distinct pathological mechanisms across all disease stages. Given these significant differences in disease progression, symptom manifestations, prognosis, and treatment responsiveness between the two stroke types, hemorrhagic stroke was excluded from the current study. Apart from that, since some neuromodulation techniques, such as transcranial magnetic stimulation (TMS), can modulate brain oscillatory activities to promote neuroplasticity (25), and these techniques have been found to achieve long-term effects on motor function recovery post stroke (37), patients who received TMS within one month were set as an exclusion criterion to avoid interference with EEG signals.

Outcome measures include the simplified Fugl-Meyer Assessment for motor function, the Berg Balance Scale, the Fugl-Meyer Assessment for sensory function, the Modified Ashworth Scale, the Modified Barthel Index, and TCM syndrome scoring scale, which are used to comprehensively evaluate the efficacy of the “simultaneous treatment of phlegm and blood stasis” acupuncture therapy based on syndrome differentiation on improving limb mobility, balance, sensation, muscle tone, activities of daily living, and TCM-related symptoms/signs in ischemic stroke patients with hemiplegia. Resting-state EEG is highly sensitive to cerebral metabolic and neurological functional abnormalities, serving as an important method for assessing post-stroke brain function (38). Commonly used frequency bands in EEG include alpha, beta, theta, and delta waves. Alpha wave activity during rest reflects the level of consciousness and visual attention (39). With a frequency range of 8–13 Hz, alpha waves are brain rhythms involved in various functions, ranging from sensorimotor processing to memory formation (40). Research indicates a linear relationship between reduced synchrony of alpha wave oscillations among brain regions and cognitive/motor function impairments in stroke patients (41). Beta waves, oscillating at 13–30 Hz, are crucial rhythms in cortical activity, closely related to motor control and sensorimotor integration (42), with their generation involving the synergistic action of pyramidal and inhibitory neurons (43). Research has found that beta wave coherence can serve as a marker for cortical functional plasticity after stroke (42). Delta waves are low-frequency waves with frequencies ranging from 1–4 Hz, and can be detected during slow-wave sleep and execution of cognitive tasks (44). Theta waves, with frequencies in the 4–8 Hz range, have been extensively studied in emotional and cognitive domains. Recent research has found that perturbation during seated movements can evoke differential theta-band responses in the anterior cingulate cortex and supplementary motor areas (45). In this study, we will employ EEG to record and analyze the frequency, amplitude, and spectral power of delta, theta, alpha, beta waves. We will designate the M1 region as the seed region and calculate the coherence of alpha and beta waves between the seed region and other regions. By observing changes in neuronal oscillation frequencies, electrical activity intensity, and functional connectivity across different brain regions before and after treatment, we aim to explore the neurophysiological mechanisms, by which acupuncture improve limb function in stroke patients, and screen EEG-based biomarkers with predictive value.

There are still some limitations in the design of this study: (1) the study does not restrict the infarction sites of included patients to the same hemisphere or specific locations, leading to increased heterogeneity in EEG signals; (2) the study duration is short, and there is a lack of follow-up observation after treatment course, making it difficult to ascertain the long-term efficacy of acupuncture therapy; (3) due to the treatment characteristics of acupuncture, it is challenging to implement blinding for acupuncturists and patients; (4) a multicenter study is not designed, making it impossible to verify the general applicability of the study across different populations and medical conditions. Hence, based on this study, we will further conduct multicenter, large-sample, long-term follow-up clinical trials for ischemic stroke patients in recovery stage.

Collectively, the primary objective of this clinical trial is to evaluate the efficacy and safety of the “simultaneous treatment of phlegm and blood stasis” acupuncture therapy in improving limb motor impairment in ischemic stroke patients with hemiplegia using functional scales, while exploring its underlying electrophysiological mechanisms through EEG. By conducting this clinical study, we expect to provide reliable scientific evidence for the clinical application of acupuncture therapy featuring characteristic acupoint combinations based on TCM syndrome differentiation in the field of neurological rehabilitation for ischemic stroke.

## 4 Trial status

This trial is now recruiting participants.
