# Supplementary material for: Therapeutic efficacy of acupuncture on motor dysfunction in ischemic stroke patients with hemiplegia and its EEG characteristics: protocol for a randomized, sham-acupuncture controlled, assessor-and-statistician-blinded trial
Source: Front Neurol. 2025 Sep 26;16:1653873. doi: 10.3389/fneur.2025.1653873 (PMC12512167; doi:10.3389/fneur.2025.1653873)
Supplement: Supplementary file 2 [file Table_2.docx]

Table S2. Standards for Reporting Interventions in Clinical Trials of Acupuncture( STRICTA )

| Section/topic | | Item number | | Checklist item | Report/  Not |
| --- | --- | --- | --- | --- | --- |
| 1.Acupuncture rationale | | | | | |
|  | 1a | | Style of acupuncture (eg, Traditional Chinese Medicine, Japanese, Korean, Western medical, Five Element, ear acupuncture, etc) . | | Section 2.10.2 “Acupuncture treatment” |
|  | 1b | | Reasoning for treatment provided, based on historical context, literature sources and/or consensus methods, with references where appropriate . | | Section 1 “Introduction” |
|  | 1c | | Extent to which treatment was varied . Not applicable | | |
| 2.Details of needling |  | |  | |  |
|  | 2a | | Number of needle insertions per subject per session (mean and range where relevant) | | Section 2.10.2 “Acupuncture treatment” |
|  | 2b | | Names (or location if no standard name) of points used (uni-/bilateral) . | | Section 2.10.2 “Acupuncture treatment” Table 2 |
|  | 2c | | Depth of insertion, based on a specified unit of measurement or on a particular tissue level . | | Section 2.10.2 “Acupuncture treatment” Table 2 |
|  | 2d | | Responses sought (eg, *de qi* or muscle twitch response) . | | Section 2.10.2 “Acupuncture treatment” |
|  | 2e | | Needle stimulation (eg, manual or electrical) . | | Section 2.10.2 “Acupuncture treatment” |
|  | 2f | | Needle retention time . | | Section 2.10.2 “Acupuncture treatment” |
|  | 2g | | Needle type (diameter, length and manufacturer or material) . | | Section 2.10.2 “Acupuncture treatment” |
| 3.Treatment regimen |  | |  | |  |
|  | 3a | | Number of treatment sessions . | | Section 2.10.2 “Acupuncture treatment” |
|  | 3b | | Frequency and duration of treatment sessions . | | Section 2.10.2 “Acupuncture treatment” |
| 4.Other components of  treatment |  | |  | |  |
|  | 4a | | Details of other interventions administered to the acupuncture group (eg, moxibustion, cupping, herbs, exercises, lifestyle advice) . | | Section 2.10.1 “Conventional treatment” |
|  | 4b | | Setting and context of treatment, including instructions to practitioners, and information and explanations to patients . | | Section 2.10.1 “Conventional treatment” |
| 5.Practitioner  background |  | |  | |  |
|  | 5 | | Description of participating acupuncturists (qualification or professional affiliation, years in acupuncture practice, other relevant experience) . | | Section 2.10.1 “Conventional treatment” |
| 6.Control or comparator  interventions |  | |  | |  |
|  | 6a | | Rationale for the control or comparator in the context of the research question, with sources that justify the choice(s). | | Section 2.10.3 “Sham acupuncture treatment” |
|  | 6b | | Precise description of the control or comparator. If sham acupuncture or any other type of acupuncture-like control is used, provide details as for items 1-3 above. | | Section 2.10.3 “Sham acupuncture treatment”, Figure 3 |

This checklist should be read in conjunction with the explanations of the Standards for Reporting Interventions in Clinical Trials of Acupuncture items.
